# Selenium added unripe *carica papaya* pulp extracts enhance wound repair through TGF-β1 and VEGF-a signalling pathway

**DOI:** 10.1186/s12906-015-0900-4

**Published:** 2015-10-15

**Authors:** Abdulrazaq Bidemi Nafiu, Mohammad Tariqur Rahman

**Affiliations:** Faculty of Science, International Islamic University Malaysia, Bandar Indera Mahkota, Kuantan 25200 Pahang, Malaysia; Faculty of Dentistry, University Malaya, Kuala Lumpur, 50603 Malaysia

**Keywords:** Collagen synthesis, Transforming growth factor (TGF), Vascular endothelial growth factor (VEGF), Polymorphonuclear neutrophil, Fibroblasts, Wound repair, Inflammation

## Abstract

**Background:**

Increased wound healing efficiency by Se^2+^ added *Carica papaya* L. (Caricaceae) fruit extract was linked to increased antioxidant and anti-inflammatory responses during healing.

We investigated the impact of Se^2+^ or Zn^2+^ added papaya water (WE) and phosphate-buffered saline (PE) extracts on cells recruitment and bio-molecular alterations on days 4 and 10 post wounding in an *in vivo* excision wound.

**Methods:**

Excision wounds were created on the dorsum of Sprague Dawley rats and treated topically twice/day with 20 μL of PE and WE (5 mg extract/mL), 0.5 μgSe^2+^ added PE and WE (PES and WES), or 100 μMZn^2+^ added PE and WE (PEZ and WEZ). Deionised water (negative) and Solcoseryl (positive) were applied on the control groups. Histochemical and biochemical assays were used to evaluate cellular and bio-molecular changes in the wound.

**Results:**

PES (PE + 0.5 μg Se^2+^) only increased significantly (*p* < 0.05) wound total protein content (95.14 ± 1.15 mg/g tissue *vs* positive control; 80.42 ± 0.86 mg/g tissue) on day 10 post wounding. PES increased significantly (*p* < 0.05) the number of fibroblasts/high power field (HPF) (75.60 ± 9.66) but decreased significantly (*p* < 0.05) the number of polymorphonuclear leukocytes/HPF (59.20 ± 12.64) in the wound compared to positive control (50.60 ± 12.58 fibroblasts/HPF, 101.00 ± 27.99 polymorphonuclear leukocytes/HPF) on day 4. Similar results were recorded for WES. PES demonstrated increased neovascularization, TGF-β1 and VEGFA expressions at day 4 and increased collagen at day 10.

**Conclusion:**

Papaya extract improved wound repair by increasing fibroblasts recruitment and reducing polymorphonuclear leukocytes infiltration through early transient expressions of TGF-β1 and VEGFA at the wound area. The processes were amplified with Se^2+^ addition.

**Electronic supplementary material:**

The online version of this article (doi:10.1186/s12906-015-0900-4) contains supplementary material, which is available to authorized users.

## Background

Wound healing property of *Carica papaya* L (papaya) has been attributed to its high content of cystein endopeptidase, mineral nutrients and vitamins [1–3]. Wound healing potential of papaya based extracts is mainly linked to its anti-inflammatory and anti-oxidant properties [4, 5]. We reported improved wound healing by unripe *C. papaya* when selenium (Se^2+^) was added to the extract. This was linked to increased antioxidant and anti-inflammatory responses during healing [5].

Wound healing involves a cascade of events through overlapping phases which include inflammation, repair or proliferation, and tissue remodelling [6]. The inflammatory phase is characterized by the presence of polymorphonuclear leukocytes (PMNLs), while the repair phase is characterised by fibroblast migration and proliferation, new blood vessel formation, protein synthesis, collagen deposition, epithelialisation, and wound contraction [7].

Balance in the inflammatory phase is crucial for increased cellular activities and angiogenesis under the influence of cytokines and growth factors, mainly vascular endothelial growth factor (VEGF) [8]. Again, transforming growth factor (TGF-β), produced by macrophages dictates fibroblasts recruitment to the wound site at early wound repair phase. Fibroblasts synthesize ground substances i.e., components of extracellular matrix on which collagens are deposited [9]. Hexosamines (HAM) and hexuronic acids (HUA) are primary molecules of ground substances which play significant role in re-organization and stabilization of collagen fibres [9]. Altogether, these contribute to efficient wound healing.

In the present study, the impact of Se^2+^ or Zn^2+^ added papaya extracts on the events at late inflammatory phase and early repair phase in an *in vivo* excision wound model is reported. Notably, Se^2+^ is essential for cell growth and differentiation by controlling redox-sensitive molecules [10]. While Zn^2+^ is an essential component of intracellular signalling pathways for cell proliferation [11]. Finally, we proposed how Se^2+^ addition to papaya extract enhances transient expression of TGF-β and VEGFA for improved wound repair.

## Methods

### Ethics statement

All handling and management procedures were carried out in accordance with the guidelines for the care and use of laboratory animals of International Islamic University Malaysia (IIUM) and approved by IIUM research ethics committee [Reference: IIUM/305/20/4/10].

### Fruit collection and extract preparation

Fully developed (matured) unripe papaya fruit, with white pulp and green skin (initial ripening stage) at 12 weeks after anthesis, was collected from a farm at Jabatan Pertanian, Perak, Malaysia. The fruit was identified and authenticated by Dr Nurziana of Herbal Laboratory, Faculty of Pharmacy, International Islamic University Malaysia (IIUM). Voucher specimen (no. Eiium 34) was deposited at the herbarium of the Faculty of Pharmacy, IIUM for future reference. Small pieces of the pulps were homogenized (1:3 w/w) in either sterile phosphate-buffered saline (PBS) or deionized water (dH_2_O); gently shaken at 37 °C for 8 h, centrifuged at 200 × g for 30 min at 4 °C and finally freeze-dried [3]. The freeze drying involves the complete removal of water from the pre-frozen extracts at a very low temperature and pressure until it become dried powder (lyophilised). Freeze drying preserves the chemical integrity, potency and effectiveness of the extract. The resulting solid powder form can be handled with ease for analytical purposes. The freeze-dried extracts were prepared for topical application on the animals’ excision wound as described previously [5]. Briefly, the preparations (20 μL) were used for the treatment. To obtain 0.5 μg Se^2+^ in 20 μL of treatment, a hydrous Na_2_SeO_3_ (172.94 g/mole) was used as the Se^2+^ source compound. A stock solution of Na_2_SeO_3_ (1.095 mg/mL) was prepared and diluted to 20 mL (54.75 μg/mL) which is equivalent to 0.5 μgSe^2+^/20 μL of the solution. The Na_2_SeO_3_ solution (54.75 μg/ml) was used to prepare papaya PBS and water extracts (5 mg/mL) used for the treatment. Double concentration of the stock solution was used to prepare the 1 μgSe^2+^/20 μL of the solution.

Separately, 100 and 200 μM Zn^2+^ from ZnSO_4_ solution were used to prepare the PBS and water extracts (5 mg/mL) of papaya. The resulting preparations (20 μL) were used for the topical treatment.

### Animals

Female Sprague Dawley rats weighing 200 ± 20 g (purchased either from UKM or USM, Malaysia) were used for wound induction. They were kept separately in cages for 7 days for acclimatization and fed with standard rat chow, filtered tap water and maintained under standard housing conditions (room temperature 24-27 °C and humidity 60–65 % with 12:12 light: dark cycles), before use.

### Wound induction and treatment

Uniform circular full thickness excision wound was created at the shaved dorsal region using 6 mm biopsy punch under light ethyl ether anesthesia. One wound per animal was induced to analyze the number of PMNL and fibroblasts; collagen deposition; neovascualrization; TGF-β and VEGFA expression. Same wound tissue sample (section) from each animal was used to analyze all parameters. Four full thickness excision wounds (6 mm in diameter) were inflicted on each rat using 6 mm biopsy punch under light ethyl ether anesthesia. Two wounds were created on both sides of the paravertebra region of the animals’ dorsum (the two wounds on both sides were separated by 2.5 cm and are 2 cm away from the spine) in the second experiment to analyze total protein, hydroxyproline (HOP), HUA and HAM. Rats showing any sign of skin infection or abnormal skin appearance, were excluded from the study before they were randomly assigned [5] to different experimental groups (*n* = 5 for each group).

Wounds were left undressed to the open environment while being treated with either PBS extracts (PE) or water extract (WE) with or without Se^2+^/Zn^2+^ dissolved in dH_2_O: 5 mg/ml WE, 5 mg/ml PE, 5 mg/ml PE + 0.5 μgSe^2+^ (PES), 5 mg/ml WE + 0.5 μgSe^2+^ (WES), 5 mg/ml PE + 100 μMZn^2+^ (PEZ), 5 mg/ml WE + 100 μMZn^2+^ (WEZ). Solcoseryl (a protein-free haemodialysate from calf’s blood) ointment and dH_2_O were used as positive (PC) and negative (NC) controls respectively. All wounds were treated topically in the morning and evening hours (twice/day).

### Wound tissue sample collection

The tissue samples were collected at day 4 i.e., at late inflammatory phase of wound healing and at day 10 i.e., at early wound repair phase from different animals. Wound samples were excised using scissors to ensure sufficient and regular amount of granulation tissue and the surrounding wound margin [12]. Animals were sacrificed by deep ethyl ether anesthesia before wound tissue samples were collected.

### Hematoxylin and eosin (H&E) staining

Wound tissues, fixed in 4 % paraformaldehyde for 12 h and embbeded in wax, were cut (5 μm) using rotary microtome (Leica, Biosystems). The tissue sections mounted onto Superfrost Plus slides (Fisher Scientific) were stained in H&E following standard protocol. Histological examination was performed morphometrically in high power field (HPF; 400× magnification) using Nikon light microscope (Nikon, Tokyo, Japan) with in-built camera attached to a monitor. Number of fibroblasts and PMNL was counted per HPF in 5 fields with NIS-element D software (Nikon, Tokyo, Japan): 2 fields laterally at the adjacent epidermis, 2 fields laterally deep in the dermis and 1 field in the center of the wound area. Counting was blind to the experimental design and performed on 5 sections from each group.

### van Gieson Staining

Mixture of saturated aqueous solution of picric acid and 1 % aqueous solution of acid fuchsin was used to visualize nuclei (stains brownish black to black), collagen (stains pink or deep red) and smooth muscle, cytoplasm, RBC and fibrin (stains yellow) [13]. Pink or red collagen stain intensity and small blood vessels yellow stained area were quantified using semi quantitative analysis as previously described [14]. The colour stain was scored on a 4 point scale (−, +, ++ and +++). The sign + or - represent presence or absence respectively.

### Immunohistochemical staining

Sections were deparaffinised with xylene, dehydrated in graded concentrations of ethanol and then antigen retrieval was performed using 0.1 % trypsin (Invitrogen, Carlsbad, CA) for 20 min at 37 °C. Tissue sections were treated with peroxidase blocking solution (Dako, REAL™ EnVision Detection System) and incubated with antibodies against anti TGF-β1 and VEGFA (Abcam) for 30 min at 37 °C. Thereafter, the sections were washed in Tris buffered saline containing 0.05 % (v/v) Tween 20; 3, 3’ diaminobenzidine (DAB) solution was used as the chromogen substrate, and hematoxylin was used as a counterstain.

### Wound protein estimation

Wound tissue samples were homogenized 5:1 (w/v) in an ice-cold isotonic 0.01 mol/L sodium phosphate buffer (pH 7.4); centrifuged for 5 min at 12,000 × g at 4 °C and the supernatant was used to determined protein concentration following the Bradford assay and using bovine serum albumin as standard.

### Estimation of wound hydroxyproline (HOP) content

Pre-weighed wound tissue samples were completely dried in oven and hydrolyzed in 5 N HCl for 12 h at 130 °C in sealed glass tubes. HOP content was determined using hydrolysate equivalent to 0.5 mg of wound samples as described earlier [15]. Colorimetric assay against HOP standards was performed and absorbance was read at 560 nm in Lambda 25 UV/Vis Spectrophotometer (Perkin Elmer, MA, USA).

### Estimation of wound hexuronic acid (HUA) content

The tissue was digested with papain (10 mg/g dry weight of tissue) in 0.5 M acetate buffer (20 mL per gram of tissue) pH 5.5, containing 5 μM cysteine and 5 μM di-sodium salt of EDTA at 65 °C for 24 h. An aliquot of this digest was hydrolysed followed by the spectrophotometric estimation of the HUA estimation in the samples [16].

### Estimation of wound hexosamine (HAM) content

Wound tissue samples were completely dried in oven at 60 °C and ground to powder. The HAM content was determined at 530 nm following the established protocol [17]. A correction factor of 0.829 was used to calculate and express amount of free HAM using the equation: HAM (mg) = (A_Sample_/A_STD_) × (0.05 × 0.829) [A_Sample_ = Absorbance of the samples, A_STD_ = Absorbance of the standard]. Glucosamine hydrochloride (0.05 mg) was used as a standard. Values were corrected to the initial wound tissue dry weight.

### Statistical analysis

Data were analyzed using the statistical software SPSS (version 17; SPSS, Inc.). Data were expressed as mean ± standard error of mean (SEM). One way-Anova was used to analyse all data and Tukey’s post-hoc test was used to find the mean differences within the groups at different time interval. Data were considered statistically significant at *p* < 0.05.

## Results

### Se^2+^ added extracts increases protein content in wound tissue

On day 10 post wounding, treatment with PES only, resulted in significant (*p* < 0.05) increase in the total protein content of the wound tissue (95.14 ± 1.15 mg/g tissue) as compared to the treatment with PC (80.42 ± 0.86 mg/g tissue). In contrast, Zn added extracts did not show any significant changes in the wound total protein content on day 4 and day 10 post wounding as compared to NC (19.43 ± 0.94 mg/g tissue day 4, 51.52 ± 1.55 mg/g tissue day 10) (Table 1).

**Table 1:** Changes in biochemical markers of wound healing at late inflammatory phase (day 4) and wound repair phase (day 10) during healing

Group	Day	Total protein	Hydroxyproline	Hexuronic acid	Hexosamine
		(mg/g tissue)	(μg/mg tissue)	(mg/g tissue)	(mg/g tissue)
NC	4	19.43 ± 0.94	8.47 ± 1.50	20.71 ± 4.36	12.54 ± 4.53
	10	51.52 ± 1.55	25.35 ± 1.04	5.82 ± 1.28	4.59 ± 1.57
PC	4	36.68 ± 1.06^* *** ****^	14.24 ± 1.31	49.39 ± 4.71^*^	28.41 ± 4.07
	10	80.42 ± 0.86^* *** ****^	40.62 ± 0.60^* ****^	8.65 ± 2.03	10.55 ± 2.91
PE	4	23.47 ± 1.38	12.06 ± 2.97	53.41 ± 6.21^* ****^	30.31 ± 6.11
	10	61.70 ± 1.37^* ****^	37.74 ± 1.24^* ****^	8.70 ± 1.70	10.12 ± 2.78
WE	4	23.18 ± 2.03	11.41 ± 2.70	25.45 ± 5.32	14.86 ± 3.60
	10	53.43 ± 1.50	27.25 ± 1.03^*^	6.30 ± 1.83	5.62 ± 1.45
PES	4	39.47 ± 2.05^* *** ****^	20.07 ± 3.19^*^	60.84 ± 6.08^* ****^	35.23 ± 4.95^*^
	10	95.14 ± 1.15^* ** *** ****^	55.15 ± 1.06^* ** *** ****^	18.12 ± 3.32^*^	18.21 ± 3.03^* ****^
WES	4	37.94 ± 1.15^* *** ****^	19.80 ± 2.51^*^	53.81 ± 7.36^* ****^	28.34 ± 3.87
	10	85.35 ± 2.11^* *** ****^	52.61 ± 1.02^* ** *** ****^	16.62 ± 4.88	16.51 ± 4.08
PEZ	4	21.31 ± 1.41	8.97 ± 1.93	26.47 ± 3.32	18.90 ± 3.21
	10	56.04 ± 1.48	30.52 ± 1.23^*^	6.85 ± 1.92	6.66 ± 2.12
WEZ	4	20.57 ± 1.60	10.83 ± 1.31	30.59 ± 6.27	20.06 ± 4.16
	10	56.97 ± 1.70	31.32 ± 1.34^*^	5.24 ± 1.64	6.53 ± 2.25

Values are expressed as mean ± SEM (*n* = 5). ^*, **, ***^ and ^****^ represent significantly increased (*p* < 0.05) wound content as compared to NC, PC, PE and WE respectively on the respective days

### Se^2+^ added extracts increases HOP, HUA and HAM content

Treatment with PES and WES resulted in significant (*p* < 0.05) increase in HOP content (20.07 ± 3.19 μg/mg tissue and 19.80 ± 2.51 μg/mg tissue respectively) on day 4 compared to NC (8.47 ± 1.50 μg/mg tissue) and significantly (*p* < 0.05) increase HOP content (55.15 ± 1.06 μg/mg tissue and 52.61 ± 1.02 μg/mg tissue respectively) on day 10 compared to NC (25.35 ± 1.04 μg/mg tissue), PC (40.62 ± 0.60 μg/mg tissue), PE (37.74 ± 1.24 μg/mg tissue) and WE (27.25 ± 1.03 μg/mg tissue). Treatment with PEZ and WEZ also resulted in significant (*p* < 0.05) increase in HOP content (30.52 ± 1.23 μg/mg tissue and 31.32 ± 1.34 μg/mg tissue respectively) on day 10 compared to NC (25.35 ± 1.04 μg/mg tissue) (Table 2). HUA content was significantly increased (*p* < 0.05) after treatment with PE (53.41 ± 6.21 mg/g tissue), PES (60.84 ± 6.08 mg/g tissue) and WES (53.81 ± 7.36 mg/g tissue) compared to NC (20.71 ± 4.36 mg/g tissue) and WE (25.45 ± 5.32 mg/g tissue) on day 4 and only after treatment with PES (18.12 ± 3.32 mg/g tissue) compared to NC (5.82 ± 1.28 mg/g tissue) on day 10. Treatment with only PES resulted in significantly increased (*p* < 0.05) HAM content (35.23 ± 4.95 mg/g tissue and 18.21 ± 3.03 mg/g tissue) both on day 4 and day 10 respectively compared to NC (12.54 ± 4.53 mg/g tissue day 4 and 4.59 ± 1.57 mg/g tissue day 10) (Table 1).

**Table 2:** Changes in collagen synthesis and vascularisation during wound healing at late inflammatory phase (day 4) and wound repair phase (day 10)

Group	Collagen synthesis		Vascularization	
	Day 4	Day 10	Day 4	Day 10
NC	-	++	-	-
PC	+	++	-	+
PE	+	++	++	++
WE	-	++	-	-
PES	+	+++	++	+++
WES	+	+++	++	+++
PEZ	+	++	+	++
WEZ	+	+	-	+

### Se^2+^ added extracts reduced PMNL infiltration but enhanced fibroblasts number

**Fig. 1:**
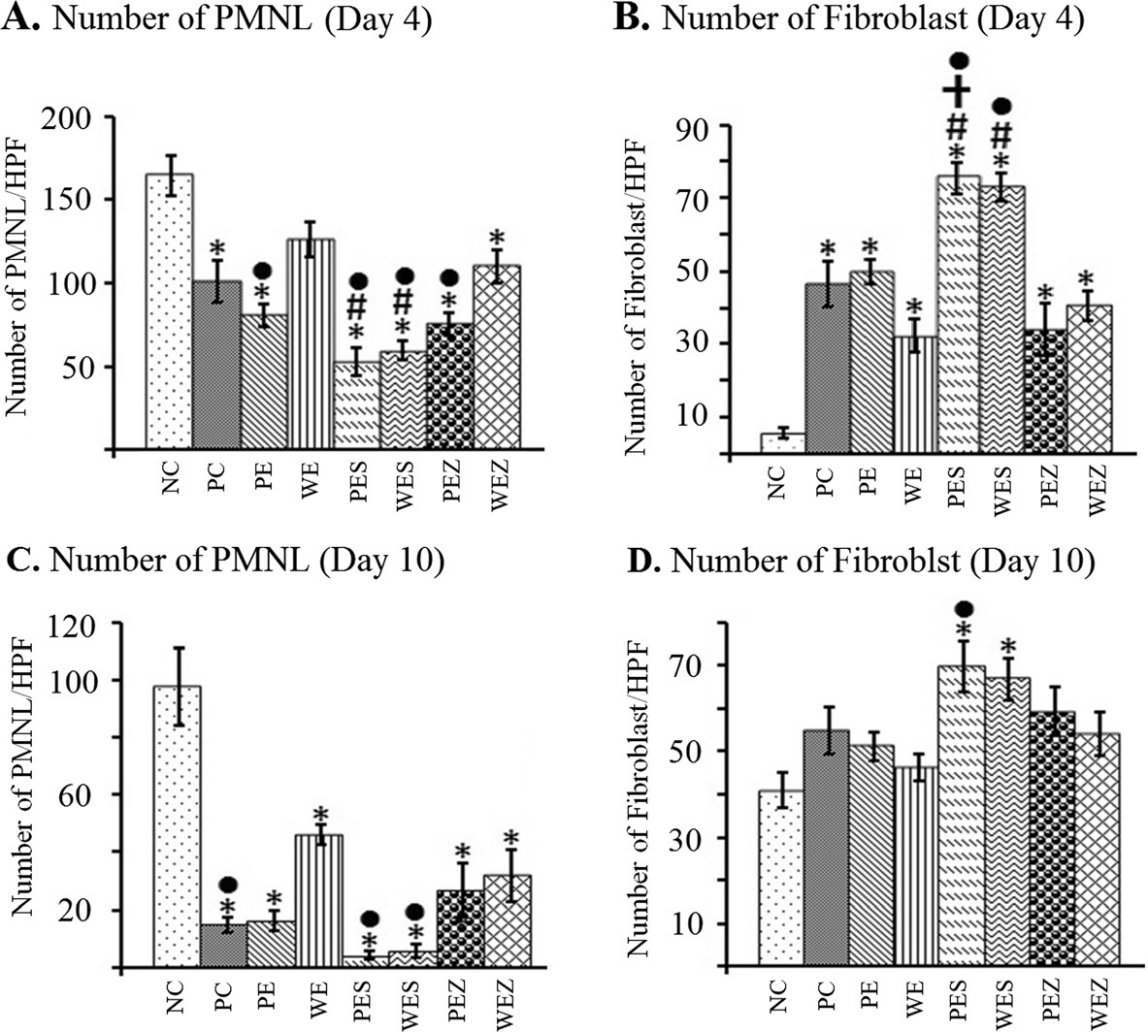
Effect of Zn^2+^ or Se^2+^ added *papaya* extracts on PMNL and fibroblast recruitment. On day 4, wounds treated with PE, WE, PEZ and WES showed reduced number of PMNL (**a**) and increased number of fibroblasts (**b**). On day 10, wounds treated with PE, WE, PEZ, and WEZ showed reduced number of infiltrating PMNL (**c**) with increased number of fibroblasts (**d**). *, #, † and ● show significant decrease of PMNL and increase of fibroblasts when compared to NC, PC, PE and WE respectively. (*p* < 0.05). [*n* = 5 per group]

The mean number of PMNL/HPF in wound tissue was significantly (*p* < 0.05) lower both on day 4 (Fig. 1a) and on day 10 (Fig. 1c) in all groups except WE (126.00 ± 23.53 day 4, 80.80 ± 31.13 day 10) compared to NC (164.40 ± 25.93 day 4, 97.60 ± 30.44 day 10). However, treatment with only PES (59.20 ± 12.63) and WES (52.80 ± 18.81) resulted in significant reduction (*p* < 0.05) of the mean number of PMNL/HPF compared to NC (164.40 ± 25.93), PC (101.00 ± 27.99) and WE (126.00 ± 23.53) on day 4 (Fig. 1a).

The mean number of fibroblast/HPF was significantly (*p* < 0.05) higher on day 4 in all treatment groups compared to NC (5.60 ± 3.21). However, significantly increased (*p* < 0.05) number of fibroblasts/HPF was observed on day 4 in PES (75.60 ± 9.66) treated only group compared to that of PC, PE and WE groups (50.60 ± 12.58, 50.00 ± 7.45 and 12.40 ± 5.94 respectively) (Fig. 1b). On day 10, the mean number of fibroblasts/HPF was significantly higher (*p* < 0.05) when treated with PES (69.60 ± 13.07) as compared to NC (40.80 ± 9.09) (Fig. 1d).

On day 4, wound tissue sections from all groups exhibited disrupted wound-dermis interface, with the absence of overlying epithelia (Additional file 1: Figure S1A). Treatment with PES exhibited a more organised dermis with firm epidermis which interdigitate with the underlying dermis consistent with a moderate fibrogenic response determined by fibroblasts number at the wound site (Additional file 1: Figure S1B).

### Se^2+^ added extract enhanced collagen synthesis and vascularization

Collagen deposition was detected on day 4 in all groups except NC and WE treated groups (Table 2, Additional file 2: Figure S2A). However, based on the semi quantitative scoring, collagen deposition was highest in PES and WES treated groups on day 4 (Table 2, Additional file 2: Figure S2B). On day 10, marked increase in pink or red collagen stain intensity was observed in WES and PES treated group with matured and well aligned collagen fibres (Additional file 2: Figure S2B). On day 4, appearance of new blood vessels was detected in PE, PES, WES and PEZ treated groups (Table 2, Additional file 2: Figure S2A). Again, on day 10, neovascularization was highest in PES and WES treated groups (Table 2, Additional file 2: Figure S2B).

### TGF-β1 and VEGFA expression in response to Se^2+^ added extracts

**Fig. 2:**
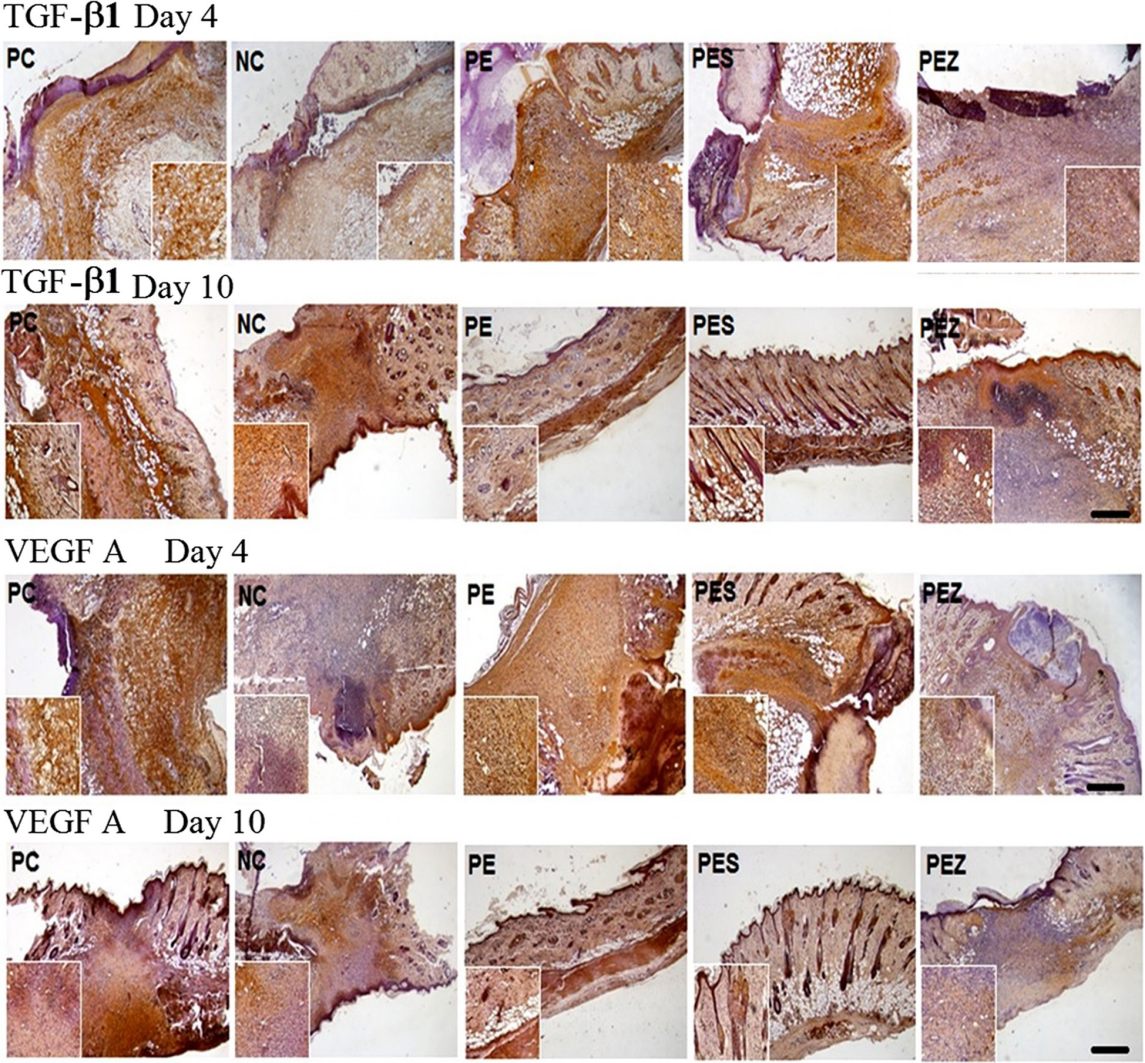
Effect of Zn^2+^ or Se^2+^ added *papaya* extracts on expression of TGF-β1 and VEGF A at the wound area. A marked increase in TGF-β1 expression in the wound area was observed only at day 4 (row 1) unlike at day 10 (row 2) after treatment with PC, PE and PES as compared to the NC and PEZ. A marked increase in VEGFA expression was observed in the wound area at day 4 (row 3) after treatment with either PC, PE or PES. Expression of VEGFA was similar in the wound at day 10 (row 4) for all treatment groups (i.e., PE, PES, PEZ and NC). [*n* = 5 per group; Scale bar = 5 μm]

Treatment with the PE, PES and PC were shown to increase TGF-β1 expression on day 4 compared to NC and PEZ (Fig. 2 row 1). On day 10, TGF-β1 expression remains low in PEZ treated group (Fig. 2 row 2). The expression of VEGF-A was increased in the PC, PE and PES treated group on day 4 as compared to NC and PEZ treated groups (Fig. 2 row 3). There was no marked difference in expression of VEGFA in the PE, PES and PEZ treated group as compared to the NC group on day 10 (Fig. 2 row 4).

## Discussions

Green unripe papaya and the latex produced from it are rich sources of the four cysteine endopeptidases enzymes namely papain, chymopapain, glycyl endopeptidase and caricain. Therapeutic potential of papaya has been attributed to it endopeptidase enzyme content. Papain, chymopapain, and glycylendopeptidase enzymes are the most abundant enzymes in the unripe pulp and latex which are consistently implicated as the therapeutic and wound healing agent in papaya [18–20].

Se^2+^, a cofactor for selenoproteins, prevents oxidative damage and protects redox-sensitive molecules involved in cell growth and differentiation [10, 21]. While Zn^2+^ is a well known functional and structural component of enzymes and transcription factors of the intracellular signaling pathways for cell proliferation [11]. Both Se^2+^ and Zn^2+^ are also involved in regulation of the concentrations of other elements (at the wound area) which are affected due to injury [22]. In relation to the current study, Se^2+^ supplementation was shown to improve healing of the wound caused by surgical incision, trauma, and burns [23]. Earlier we reported that Se^2+^ added unripe *C. papaya* improves healing of excision wound through enhanced anti-inflammatory and antioxidant effect [5].

The present study used female rats for the experimentation. Female rats are known to exhibit shorter period for complete wound healing and this has been linked to the circulating oestrogen level [24–26]. Though many previous studies used male rats for their various investigations on wound healing to minimize the variability that fluctuating circulating oestrogen may have on the rate of wound closure, some studies have also used female rats to achieve shorter period of experimentation, reduce the stressful handling of relatively aggressive male rats during examination of wound and extend the relevance of the research data to female gender despite the effect of fluctuating circulating oestrogen on the rate of healing [27, 28].

Depending on the type and aetiology of wound, wound healing involves inflammation (day 1–4 or more), proliferative/repair (day 5–10 or 11), and remodelling phases (day 11–21 or more) [9, 10]. Prolonged or excessive inflammatory responses delay the repair mechanism and subsequently affect overall wound healing. Earlier we reported that Se^2+^ added papaya mediates improved wound healing by reducing inflammation associated oxidative damage apparently via cyclooxygenase specific inhibition, arginine metabolism and up-regulation of antioxidant enzymes. These could be linked to the added Se^2+^ [5] as well as the papaya cysteine endopeptidases. Papaya promotes granulation tissue formation, nectrotic tissue desloughing and prevent infection during wound healing through the proteolytic and antimicrobial activities of it cysteine endopeptidases [29].

PMNL infiltration of wound area was found to be reduced in Zn^2+^ added PE/WE treated groups albeit the reduction was not as significant as Se^2+^ added PE/WE treated groups (Fig. 1a, c). Zn^2+^ added PE/WE treated groups showed significant increase in HOP on day 10 only and was by far lower compared to Se^2+^ added extract treated groups (Table 1). A significant increase in total protein and HOP was recorded at days 4 and 10 among Se^2+^ added extract treated groups. The same group exhibited mature and well aligned collagen fibers at day 10 (Additional file 2: Figure S2). Notably, wound hydroxylproline concentration is a common quantitative index for collagen synthesis and is related to wound tensile strength (breaking force) that generally increases at the later phase of healing i.e., proliferative/repair phase [3, 7]. Again, in line with this view, WES and PES exhibited significantly increased amount of HOP compared to NC, PC, PE and WE both at day 4 and day 10 (Table 2). Despite the widely reported role of Zn^2+^ as co-factor for cellular proliferation, antioxidant enzyme system and tissue repair and regeneration, its role in non-deficient state is yet to be confirmed. An optimal concentration or supplemental dose required to produce healing effect is yet to be established. Studies have confirmed that supplemental Zn^2+^ beyond or below certain critical dose produced negative effect on wound healing by altering the activation of nuclear transcription factors to increase the production of pro-inflammatory cytokines leading to immunosuppression [30]. These may account for the insignificant or little benefit of Zn^2+^ added PE/WE treatment on wound total protein and hydroxyproline respectively.

Treatment with Se^2+^ added papaya extract markedly increased fibroblasts number at day 4 and day 10 while Zn^2+^ added PE/WE showed a far less increase fibroblasts number at day 4 only (Fig. 1b, d). Fibroblasts recruitment at the wound area with reduced infiltrating inflammatory cells enhances wound contraction hence decrease time for complete wound closure [31]. Se^2+^ added PE/WE treatment only, exhibited significant increase in HUA and HAM at day 4 and day 10 (Table 1). HUA and HAM are primary components of ground substances (glycosaminoglycans and proteoglycans), which are synthesized by fibroblasts at the wound area to form provisional matrix on which collagen fibres are embedded [9]. Since fibroblasts produce pro-fibrotic and angiogenic cytokines, collagen, matrix through which other cells migrate [31] and provide mechanical support for delicate new capillaries [8, 12], increased fibroblasts recruitment at the wound area could enhance wound repair.

Consistently, neovascularization is more noticeable at day 4 both in PE and PES treated groups (Table 2). The new blood vessel at the wound area supplies more nutrient and oxygen to the fibroblasts to support subsequent production of growth factors and collagen synthesis. Thus, these results demonstrated that the degree of dermal repair is consistent with fibroblasts recruitment at the wound site and papaya extracts added with Se^2+^ enhanced dermal repair.

In the current study we have reported that PES induces early neovascularization (Table 2) and enhance fibroblasts recruitment (Fig. 1b, d) at the wound area. The increased expressions of TGF-β1 and VEGFA, in response to PES at day 4 but not at day 10 (Fig. 2) thus, suggests that Se^2+^ addition improves wound healing by inducing early transient expressions of TGF-β1 and VEGFA followed by enhanced angiogenic sprouting and increased fibroblasts recruitment during the early phase of healing. Angiogenic factor VEGFA and TGF-β play key roles in the natural cutaneous wound healing by attracting inflammatory cells, stimulating cellular proliferation, neovascularization and epithelial migration [31]. Selenium has been reported to induce expression of VEGFA in diabetic wound [32]. Selenium as low as 10^−7^ M activate aortal and capillary endothelial cells; increase proliferation of aortal cells and migration of retinal capillary cells [33].

**Fig. 3:**
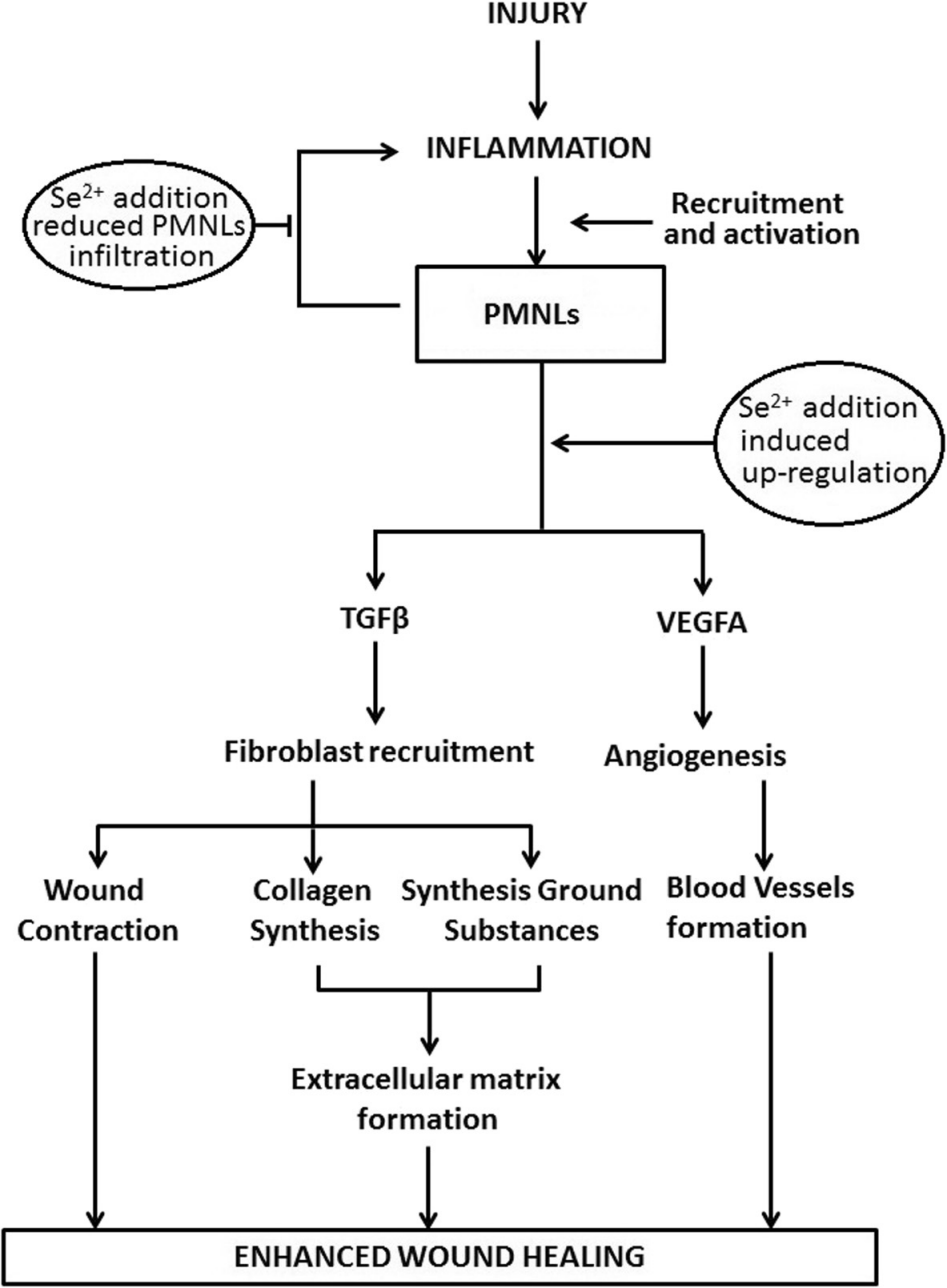
Events controlled by Se^2+^ added papaya extract in cutaneous wound healing cascade. Se^2+^ added papaya PBS extract treatment results in the transient up-regulation of (TGF-β and VEGFA at earlier phase of healing (Fig. 2) and consequently results in increased fibroblasts recruitment (Fig. 1b, d) and angiogenesis (Table 2). The expression of TGF-β in turn promotes synthesis of collagen and ground substances while expression of VEGF promotes new blood vessel formation through angiogenesis. Se^2+^ added papaya PBS extract either stops or reduces further PMNL infiltration (Fig. 1a, c) at the wound area thus subsides inflammatory reactions. All together these contribute to accelerated and improved wound healing without inflammation at later stages

Based on the observed up and down regulation of the cellular (PMNL infiltration and fibroblast recruitment, neovascularization) and biochemical events (collagen synthesis, TGF-β1 and VEGFA expression), a model is proposed (Fig. 3) to describe the possible mechanisms by which *Carica papaya* extracts enhanced cutaneous wound repair.

PE through mechanisms associated with cyclooxygenase and prostaglanding E2 inhibition [5] reduced PMNLs infiltration of wound area in the early phase of wound repair. Reduction of PMNLs infiltration by PE, accelerate the inflammatory phase which consequently expedite the onset of proliferative/repair phase. This process is potentiated by Se addition to PE.

Consistently, PE induced a transient up-regulation of TGF-β1 and VEGFA on day 4 which in turn enhanced cellular activity (proliferation, differentiation, migration, angiogenesis and neovascularisation) as evidenced in the increased fibroblasts recruitment and early appearance of new blood vessels (indicator of endothelial cell differentiation) in PE treatment. These effect was more pronounced in Se added PE.

In addition, the establishment of new blood vessels enhanced nutrient delivery to the fibroblasts and enhanced fibroblastic synthesis of provisional matrix, primary molecules in the extracellular matrix and collagen. In line with this, Se added PE exclusively enhanced the synthesis of primary molecules of ground substances i.e. HUA and HAM. PE alone showed little effect in these regard. However, hydroxyproline an index for collagen turnover was found to be markedly increased, later on day 10, with PE treatment. Concurrently, well aligned collagen fibres and reconstructed dermis and epidermis were observed later on day 10 after treatment with PE and PES especially. Collectively, the increased recruitment of fibroblasts and their bio-molcular products resulted in the significant increase in wound total protein content later on day 10 in PE treated group.

## Conclusions

Se^2+^ added papaya extracts might (i) reduce PMNL infiltration to speed up the inflammatory phase as well as (ii) induced transient up-regulation of TGF-β1 and VEGFA expression to enhance angiogenesis and fibroblast recruitment thereby enhanced cutaneous wound repair.

## Additional files

Additional file 1: Figure S1. Histology of Polymorphonuclear leukocytes (PMNL) and fibroblasts on wound tissue after topical application of Zn^2+^ or Se^2+^ added papaya extracts. PMNL (arrow) and fibroblasts (arrow head) at late inflammatory phase i.e., day 4 (A) and wound repair phase i.e., at day 10 (B) post wounding after treatment. At day 10 post wounding. Disrupted dermis with high density of infiltrating inflammatory cells, thin overlying epithelia and lower number of fibroblasts were observed in the negative control (NC) wounds. However, wounds treated with Se^2+^ (0.5 μg) added papaya PBS and water extracts (PES and WES respectively) exhibited firmly attached epithelium which interdigitate with a more organised dermis, a marked reduction of infiltrating PMNL with significant increased number of fibroblasts as compared to NC and WE. [Scale Bar = 50 μm]. (JPEG 1233 kb) Additional file 2: Figure S2. Effect of topical application of Zn^2+^ or Se^2+^ added *papaya* extracts on vascularization and collagen synthesis. Representative photomicrographs of Van Gieson staining for collagen fibers (arrow) and blood vessels (arrow head) in wound tissue sections at late inflammatory phase i.e., day 4 (A) and wound repair phase i.e., at day 10 (B) post wounding after treatment. At day 4, traces of collagen deposits were observed in all treated wounds except in WE and NC wounds. Wounds treated PE, PES and WES showed appearance of blood vessels in the dermis. Sign of regeneration and repair such as hair follicle, glands and aligned collagen fibers were absent in all groups. At day 10 (B), matured collagen fibres, glands (open arrow) and hair follicle (diamond arrow) were observed in both PES and WES treated wounds while less matured but significant collagen deposits were observed in other treated wounds. PEZ and WEZ treated wounds showed appearance of blood vessels. NC, Negative control; PC, positive control group; PE and WE were applied at 5 mg/ml; PES, PE + 0.5 μg Se^2+^; WES, WE + 0.5 μg Se^2+^; PEZ, PE + 100 μM Zn^2+^; WEZ, WE + 100 μM Zn^2+^. WE and PE is applied at 5 mg/ml; PES, PE + 0.5 μg Se^2+^; WES, WE + 0.5 μg Se^2+^; PEZ, PE + 100 μM Zn^2+^; WEZ, WE + 100 μM Zn^2+^. [Scale Bar = 50 μm]. (JPEG 1924 kb)
