# Supplementary figures and images for: Selenium added unripe carica papaya pulp extracts enhance wound repair through TGF-β1 and VEGF-a signalling pathway
Source: BMC Complement Altern Med. 2015 Oct 15;15:369. doi: 10.1186/s12906-015-0900-4 (PMC4608175; doi:10.1186/s12906-015-0900-4)

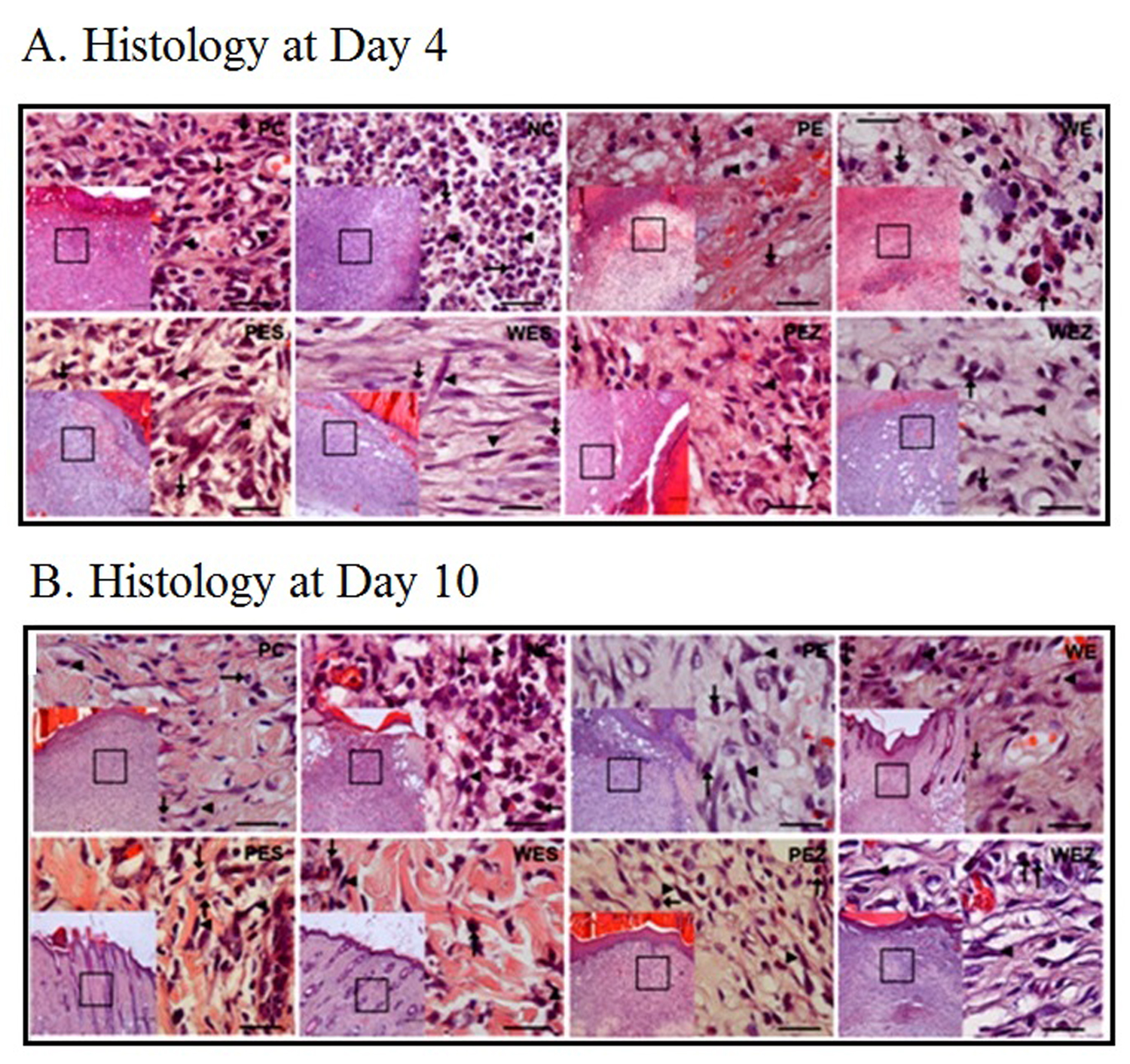

Supplement: Additional file 1: Figure S1. — Histology of Polymorphonuclear leukocytes (PMNL) and fibroblasts on wound tissue after topical application of Zn2+ or Se2+ added papaya extracts. PMNL (arrow) and fibroblasts (arrow head) at late inflammatory phase i.e., day 4 (A) and wound repair phase i.e., at day 10 (B) post wounding after treatment. At day 10 post wounding. Disrupted dermis with high density of infiltrating inflammatory cells, thin overlying epithelia and lower number of fibroblasts were observed in the negative control (NC) wounds. However, wounds treated with Se2+ (0.5 μg) added papaya PBS and water extracts (PES and WES respectively) exhibited firmly attached epithelium which interdigitate with a more organised dermis, a marked reduction of infiltrating PMNL with significant increased number of fibroblasts as compared to NC and WE. [Scale Bar = 50 μm]. (JPEG 1233 kb) [file 12906_2015_900_MOESM1_ESM.jpg]

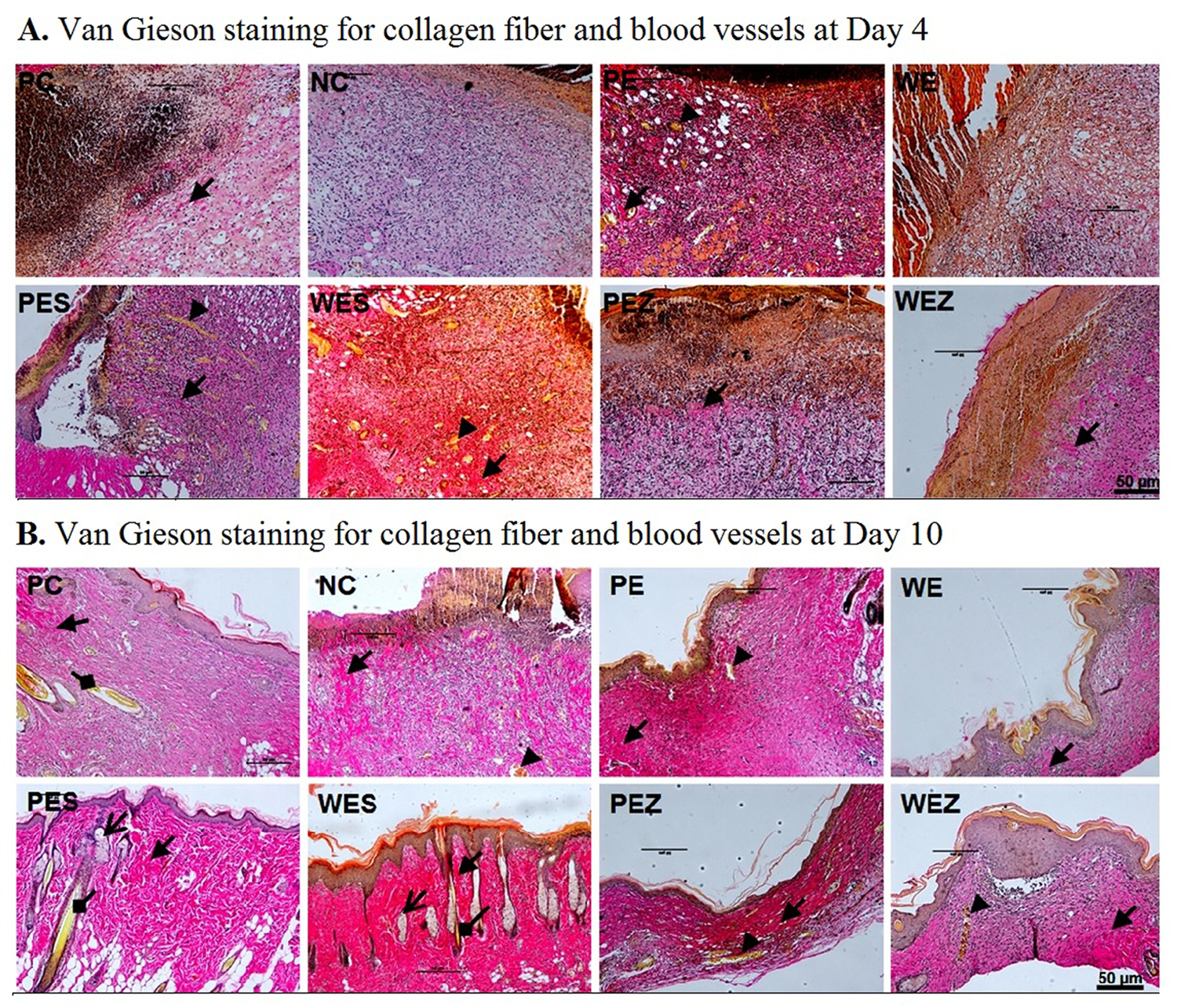

Supplement: Additional file 2: Figure S2. — Effect of topical application of Zn2+ or Se2+ added papaya extracts on vascularization and collagen synthesis. Representative photomicrographs of Van Gieson staining for collagen fibers (arrow) and blood vessels (arrow head) in wound tissue sections at late inflammatory phase i.e., day 4 (A) and wound repair phase i.e., at day 10 (B) post wounding after treatment. At day 4, traces of collagen deposits were observed in all treated wounds except in WE and NC wounds. Wounds treated PE, PES and WES showed appearance of blood vessels in the dermis. Sign of regeneration and repair such as hair follicle, glands and aligned collagen fibers were absent in all groups. At day 10 (B), matured collagen fibres, glands (open arrow) and hair follicle (diamond arrow) were observed in both PES and WES treated wounds while less matured but significant collagen deposits were observed in other treated wounds. PEZ and WEZ treated wounds showed appearance of blood vessels. NC, Negative control; PC, positive control group; PE and WE were applied at 5 mg/ml; PES, PE + 0.5 μg Se2+; WES, WE + 0.5 μg Se2+; PEZ, PE + 100 μM Zn2+; WEZ, WE + 100 μM Zn2+. WE and PE is applied at 5 mg/ml; PES, PE + 0.5 μg Se2+; WES, WE + 0.5 μg Se2+; PEZ, PE + 100 μM Zn2+; WEZ, WE + 100 μM Zn2+. [Scale Bar = 50 μm]. (JPEG 1924 kb) [file 12906_2015_900_MOESM2_ESM.jpg]
